# Transcatheter closure of postsurgical aortic pseudoaneurysms guided by three-dimensional image reconstruction: a single-centre experience

**DOI:** 10.1007/s12471-023-01784-1

**Published:** 2023-05-31

**Authors:** Romy R. M. J. J. Hegeman, Martin J. Swaans, Basak Kara, Robin H. Heijmen, Hans G. Smeenk, Leo Timmers, Uday Sonker, Patrick Klein, Jurriën M. Ten Berg

**Affiliations:** 1 Department of Cardiothoracic Surgery, St. Antonius Hospital, Nieuwegein, The Netherlands; 2 Department of Cardiology, St. Antonius Hospital, Nieuwegein, The Netherlands; 3 Department of Cardiothoracic Surgery, Radboud University Medical Centre, Nijmegen, The Netherlands

**Keywords:** False aneurysm, Pseudoaneurysm, Closure device, Transcatheter closure, Three-dimensional reconstruction

## Abstract

**Background:**

Postsurgical thoracic aortic pseudoaneurysms (PTAPs) are a potentially lethal complication after cardiac or aortic surgery. Surgical management can pose a challenge with high in-hospital mortality rates. Transcatheter closure is a less-invasive alternative treatment option for selected patients, although current experience is limited.

**Aims:**

We aimed to evaluate procedural and imaging outcomes of our first 11 cases of transcatheter PTAP closure with the use of closure devices.

**Methods:**

Patients with a high operative risk who underwent transcatheter PTAP closure at our centre from 2019 to 2021 were retrospectively included. Suitability was evaluated on preprocedural computed tomography (CT) scans and three-dimensional (3D) reconstructions. All procedures were performed in the catheterisation laboratory. Intraprocedural aortography and postprocedural CT scans with 3D reconstructions were used to evaluate PTAP occlusion.

**Results:**

Eleven consecutive patients with a high operative risk and a history of cardiac/aortic surgery who underwent transcatheter PTAP closure were included. PTAPs were predominantly located at the proximal or distal anastomosis of a supracoronary ascending aortic vascular graft or Bentall prosthesis (82%). Implanted closure devices included Amplatzer Valvular Plug III (82%), Amplatzer septal occluder (9%) and Occlutech atrial septal defect occluder (9%). No periprocedural complications occurred. After device deployment, residual flow was absent on aortography in 64% and minimal residual flow was present in 36% of patients. Subtotal or total occlusion of the PTAP on follow-up CT ranged between 45% and 73%.

**Conclusions:**

Although subtotal or total occlusion of the PTAP was found at follow-up in only 45–73% of cases, transcatheter PTAP closure guided by preprocedural 3D reconstructions can offer a valuable minimally invasive primary treatment option for patients who otherwise would face a high-risk reoperation.

**Supplementary Information:**

The online version of this article (10.1007/s12471-023-01784-1) contains supplementary material, which is available to authorized users.

## What’s new?

- Procedural and imaging outcomes of 11 high-risk cases of transcatheter postsurgical thoracic aortic pseudoaneurysm (PTAP) closure with closure devices were evaluated.
- No periprocedural complications occurred.
- After device deployment, residual flow was absent on aortography in 64% and minimal residual flow was present in 36% of patients.
- On early and latest follow-up CT, complete occlusion of the PTAP was reported in 36% of patients. Partial occlusion was achieved in 36% at early follow-up and 9% at latest follow-up.
- Although subtotal or total occlusion was accomplished in only 45–73% of our cases, transcatheter closure can offer a valuable minimally invasive primary treatment option for patients who otherwise would face a high-risk reoperation.

## Introduction

Postsurgical thoracic aortic pseudoaneurysms (PTAPs) often result from anastomotic dehiscence and are characterised by extravasation of blood contained in periarterial connective tissue [1, 2]. Although they are often asymptomatic and only detected during routine follow-up [3], a progressive increase in the size of a PTAP can cause fatal rupture, fistula formation and compression or erosion of surrounding structures [4]. Therefore, PTAPs are associated with a mortality rate up to 61% when left untreated [5]. Current guidelines state that transcatheter or surgical intervention is indicated if there are no contraindications [4]. However, depending on aetiology and location, surgical management can pose a challenge with in-hospital mortality rates between 7% and 41% [2, 6, 7]. As an alternative approach, endovascular repair with a stent graft can be performed but requires favourable anatomical features, including the presence of a proximal and distal landing zone together with adequate iliac or femoral vessels for vascular access [4]. Transcatheter coil embolisation can be performed as a stand-alone therapy for small PTAPs with narrow necks and for saccular aneurysms with a sac diameter greater than the neck in order to enable intra-aneurysmal coil packing without the risk of coil displacement [7].

**Fig. 1:**
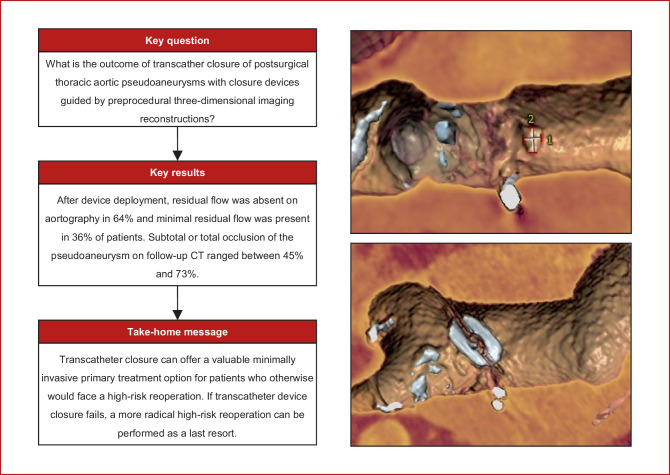
Infographic

Transcatheter closure with the use of a closure device is a less-invasive alternative treatment option for selected patients, although experience is still limited and the long-term outcome unknown [3]. Here, we present our single-centre experience with the first 11 cases of transcatheter PTAP closure, which is the largest reported series using this technique (Fig. 1).

## Methods

### Patient selection

All patients who underwent transcatheter PTAP closure between March 2019 and April 2021 were retrospectively included. All patients with a PTAP confirmed on computed tomography (CT) were discussed in our multi-disciplinary team comprising, among others, cardiac surgeons, interventional cardiologists and an imaging cardiologist. Patients were eligible for transcatheter closure if they had been rejected for surgery by the heart team because of a prohibitive surgical risk (based on extensive cardiothoracic surgical history and EuroSCORE II ≥ 5.0).

### Imaging

CT images and three-dimensional (3D) reconstructions derived from preprocedural CT images with 3Mensio Structural Heart (Pie Medical Imaging, Maastricht, The Netherlands) were used to evaluate suitability for transcatheter closure and to determine the optimal size of the plug. Intraprocedural aortography was performed to confirm correct placement of the closure device and to identify residual leakage into the PTAP.

Occlusion of the PTAP was evaluated by follow-up CT and categorised as: complete occlusion, partial occlusion (i.e. non-thrombosed PTAP remnant with > 50% reduction of preprocedural PTAP diameters for which no reintervention was required), significant residual flow (i.e. non-thrombosed PTAP remnant with < 50% reduction of preprocedural PTAP diameters for which monitoring or reintervention was required). 3D reconstructions were derived from all postprocedural CT scans.

### Procedure

Procedures were performed in the catheterisation laboratory with the patient under local anaesthesia. A sheath was inserted into the right or left femoral artery, through which a guiding catheter (6–8 Fr Amplatz Left 1, Boston Scientific, Marlborough, MA, USA) was introduced and advanced into the neck or body of the PTAP. Consequently, the closure device was positioned and deployed in the neck of the PTAP.

### Capacity of St. Antonius Hospital

The multidisciplinary team of the Heart Centre of St. Antonius Hospital Nieuwegein comprises, among others, six interventional cardiologists, one dedicated imaging cardiologist and nine cardiothoracic surgeons. Of this team, two interventional cardiologists have performed PTAP closure since 2019 in co-operation with two cardiothoracic surgeons and the imaging cardiologist. Annually, approximately 30 percutaneous aortic repair procedures are performed, including paravalvular leak closure and PTAP closure.

### Statistical analysis

Patient characteristics and surgical outcomes were analysed using IBM SPSS Statistics 26.0 (IBM Corp, Armonk, NY, USA). Procedural data are summarised per patient. Continuous pooled data are presented as mean ± standard deviation (SD) or median [interquartile range (IQR)]. Categorical outcomes were summarised as the number and percentage of patients in each category.

## Results

### Patient characteristics

Patient characteristics are shown in Tab. 1. Eleven consecutive patients who underwent transcatheter PTAP closure were included between March 2019 and April 2021. Only two patients (18%) were symptomatic: patient 1 presented with chest pain and dyspnoea during exercise and patient 3 also had complaints of dyspnoea during exercise. Six patients underwent a Bentall procedure with (*n* = 2) or without (*n* = 4) partial arch replacement and with or without concomitant coronary artery bypass grafting (CABG). In one patient isolated CABG was performed. Four patients underwent a supracoronary ascending aorta replacement (SCAR) with or without partial/total arch replacement and CABG. Time from first surgery to diagnosis of the PTAP ranged from 2 years to 27 years (Table S1, Electronic Supplementary Material). Two patients had already undergone transcatheter coil embolisation of the same PTAP without success.

**Table 1:** Baseline characteristics

Characteristic	All patients (*n* = 11)
Age (years)	73 ± 6
Male	8 (73%)
Body mass index (kg/m^2^)	25 ± 4
Atrial fibrillation	7 (64%)
Hypertension	9 (82%)
Diabetes mellitus	1 (9%)
Serum creatinine > 80 µmol/l	9 (82%)
eGFR < 60 ml/min per 1.73 m^2^	6 (55%)
Chronic obstructive pulmonary disease	0 (0%)
Previous stroke or transient ischaemic attack	2 (18%)
Symptomatic	2 (18%)
EuroSCORE II (%)	9.0 ± 6.0%

Values are mean ± SD or *n* (%)

*eGFR* estimated glomerular filtration rate

PTAPs were located at the proximal (*n* = 1) or distal (*n* = 8) SCAR or Bentall anastomosis and at the right coronary button after a Bentall procedure (*n* = 1) and right coronary ostium after isolated CABG, possibly as a consequence of recent endocarditis (*n* = 1). The measurements of the pseudoaneurysmal sac and neck on the preprocedural CT scan of each patient are summarised in Table S1 (Electronic Supplementary Material).

### Procedural information

Procedural information is presented in Tab. 2, S1. Nine patients (82%) received an Amplatzer Valvular Plug III (AVP-3) (St. Jude Medical, St. Paul, MN, USA), one patient (9%) received an Amplatzer septal occluder with a diameter of 10 mm (Abbott, Abbott Park, IL, USA) and one patient (9%) an Occlutech atrial septal defect (ASD) occluder with a diameter of 15 mm (Occlutech International AB, Helsingborg, Sweden). Sizes of implanted AVP‑3 closure devices were 6 × 3 mm (*n* = 4), 8 × 4 mm (*n* = 1), 10 × 5 mm (*n* = 3) and 14 × 5 mm (*n* = 1). No significant association was found between the neck diameter and implanted device diameter (i.e. long axis, *p* = 0.798). No periprocedural complications occurred in any patient. Anticoagulants were briefly discontinued during the periprocedural period in three cases and continued in eight patients. The peri- and postprocedural continuation of anticoagulation therapy was based on the policy of the treating physician and the preoperative indication for oral anticoagulation. Median duration of hospitalisation was 1.0 days (IQR 1.0–2.0).

**Table 2:** Periprocedural information

Characteristic	All patients (*n* = 11)
Access site	
– Right femoral artery – Left femoral artery	10 (90.9%) 1 (9.1%)
Device type	
– AVP‑3 – Occlutech ASD occluder – Amplatzer septal occluder	9 (81.8%) 1 (9.1%) 1 (9.1%)
Hospitalisation (days)	1 (IQR 1–2)
Residual flow on aortography directly after device deployment	
– No residual flow – Minimal residual flow	7 (63.6%) 4 (36.4%)
Occlusion on follow-up CT	
Short-term follow-up CT^a^ (months) – Total occlusion – Partial occlusion – Significant residual flow requiring reintervention or close monitoring	2 (IQR 2–3) 4 (36.4%) 4 (36.4%) 3 (27.3%)
Last follow-up CT^b^ (months) – Total occlusion – Partial occlusion – Significant residual flow on last CT requiring reintervention or close monitoring	11 (IQR 9–16) 4 (36.4%) 1 (9.1%) 4 (36.4%)

Values are mean ± SD, median (IQR) or *n* (%)

*ASD* atrial septal defect, *AVP‑3* Amplatzer Valvular Plug III, *CT* computed tomography, *Fr* French

^a^CT scan performed within 6 months postoperatively

^b^Second CT scan performed after first follow-up CT. A second CT scan was not available for two patients

**Fig. 2:**
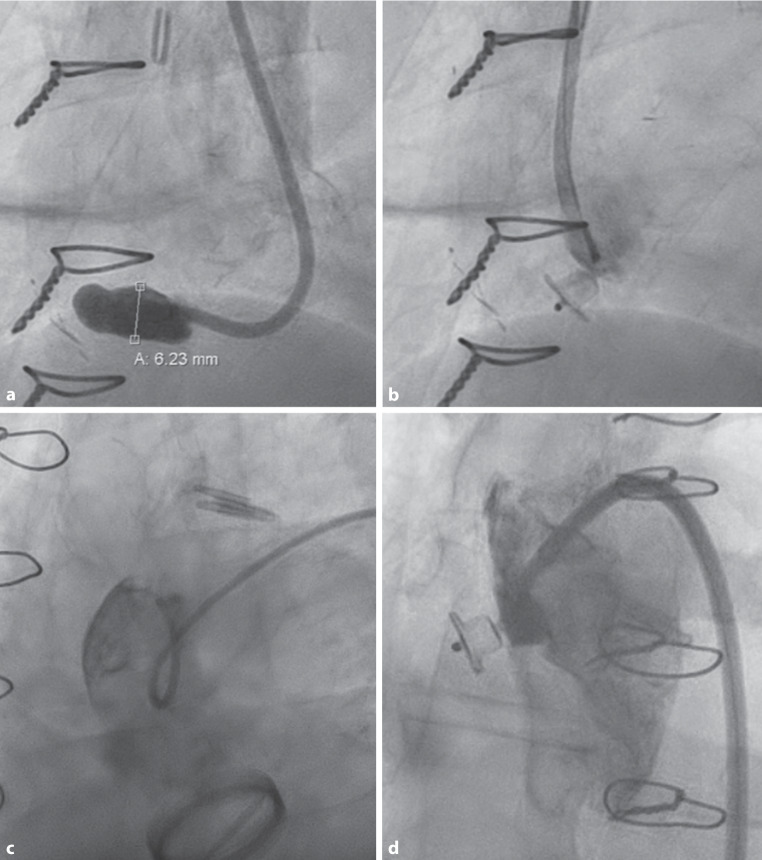
**a–d** Aortography before and after device deployment. **a** Aortography before deployment of an Amplatzer Valvular Plug III (AVP-3) device (case 4), showing flow into the postsurgical thoracic aortic pseudoaneurysm (PTAP). **b** Aortography after AVP‑3 device deployment (case 4), showing no residual flow into the PTAP. **c** Aortography before AVP‑3 device deployment (case 2), showing flow into the PTAP. **d** Aortography after AVP‑3 device deployment (case 2), showing no residual flow into the PTAP

Directly after device deployment, aortography showed complete absence of residual flow into the PTAP in seven (64%) patients and minimal residual flow in four (36%) patients (Fig. 2). Short-term follow-up CT (i.e. the first follow-up CT scan performed within 6 months postoperatively) at a median interval of 2 (IQR 2–3) months postoperatively revealed complete occlusion of the PTAP in four (36%) patients and partial occlusion in four (36%) patients. Significant residual flow was found in three (27%) patients, of whom one showed minimal residual flow on aortography directly postdeployment. Similarly, two of four patients with partial PTAP occlusion on short-term follow-up CT demonstrated minimal residual flow on aortography after device placement. All patients with partial to significant residual flow on short-term follow-up CT continued anticoagulants during the periprocedural period, including clopidogrel (*n* = 1), acenocoumarol with aspirin (*n* = 2) and direct oral anticoagulants (*n* = 2).

One or more additional follow-up CT scans were performed in nine patients, revealing complete occlusion of the PTAP in four (36%), partial occlusion in one (9%) and significant residual flow in four (36%) patients. All patients with significant residual flow on the first CT scan also had significant residual flow on the last CT scan. One patient progressed from partial occlusion to significant residual flow, but due to the multiple comorbidities it was decided to remain conservative as regards treatment of the PTAP.

Due to significant residual flow, reintervention with a second AVP‑3 was attempted 17 months postprocedure in one patient but was unsuccessful because the guiding catheter could not be advanced into the PTAP. The dimensions of the PTAP did not significantly increase over time up to the last follow-up 1 year after reintervention. The patient remained asymptomatic and is monitored yearly.

Another patient with significant residual flow but without growth of the PTAP on the first follow-up CT scan was monitored, and also remained free of symptoms. Since the PTAP diameter had increased on the second follow-up CT scan 10 months postprocedure, a second reoperation was performed despite the high surgical risk. Surgery was complicated by severe bleeding caused by injury of the old prosthesis during resternotomy due to retrosternal adhesions. A new Gelweave Valsalva graft (Terumo Aortic, Vascutek Ltd, Inchinnan, UK) was placed. Directly postsurgery, the patient developed a postanoxic encephalopathy and died shortly thereafter.

The third patient presented with syncope and chest pain 7 weeks postprocedure. The CT scan revealed rupture of the PTAP with a haematoma in the anterior mediastinum. Because of the high mortality risk of a third repeat Bentall procedure, increased technical difficulty involved in placement of a second plug and lack of an adequate landing zone for a thoracic endovascular aortic repair (TEVAR) prosthesis, conservative palliative treatment was decided upon. The patient died 4 months postprocedure.

**Fig. 3:**
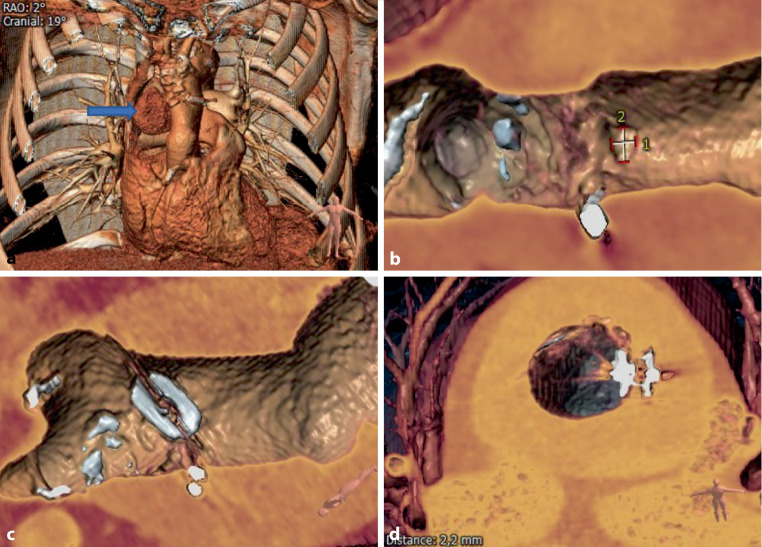
**a–d** Pre- and postprocedural 3D reconstructions in case 2. **a** Preprocedural 3D reconstruction of the thoracic cavity, showing the large postsurgical thoracic aortic pseudoaneurysm (PTAP) located right lateral to the ascending aorta (*arrow*), at the level of the distal anastomosis of the Bentall prosthesis. **b** Preprocedural 3D reconstruction of the ascending aorta: intra-aortic view, showing the opening/neck of the PTAP from the ascending aorta. **c** 3D reconstruction of the ascending aorta: intra-aortic view after placement of the AVP‑3. **d** 3D reconstruction of the ascending aorta: transverse aortic view after placement of the AVP‑3, showing closure of the PTAP

**Fig. 4:**
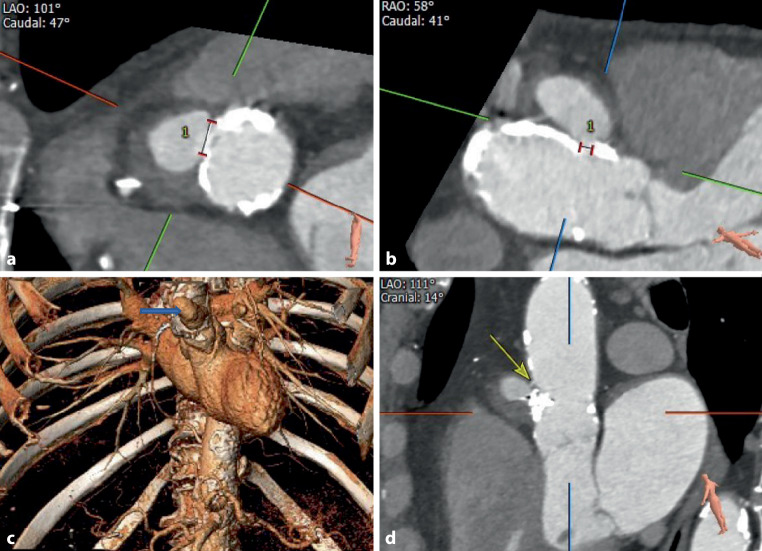
**a–d** Pre- and postprocedural 3D reconstructions in case 5. **a** Left anterior oblique (LAO) caudal view of 3D reconstruction before plug placement. *1* neck width 10.5 mm. **b** Right anterior oblique caudal view of 3D reconstruction before plug placement. *1* neck width 3.7 mm. **c** Preprocedural 3D reconstruction of the thoracic cavity, showing the large postsurgical thoracic aortic pseudoaneurysm (PTAP) (*arrow*) located at the anterior aortic root, just above the right coronary button. **d** LAO caudal view of 3D reconstruction after Amplatzer Valvular Plug III placement, revealing incomplete sealing of the PTAP (*arrow*)

Pre- and postprocedural 3D image reconstructions in a successful case are shown in Fig. 3, revealing complete closure of the PTAP after placement of an AVP‑3 device. Figure 4 demonstrates 3D imaging in an unsuccessful case with incomplete sealing.

## Discussion

Treatment options for PTAPs range from surgical repair to less invasive endovascular stent grafting and transcatheter closure with the use of coil embolisation or closure devices. Although surgical repair is accompanied by relatively high mortality rates [8], it remains the gold standard treatment given the larger amount of experience [7]. However, if the patient is deemed unsuitable for surgery because of a high perioperative mortality risk, an alternative less-invasive treatment option can be selected. Equivalently, if there is an inadequate landing zone and/or proximity to supra-aortic vessels, transcatheter closure should be preferred over a stent graft [9].

When selecting patients for percutaneous device closure, the anatomical features of the PTAP (i.e. the location and size of the neck and aneurysmal sac) need to be meticulously evaluated. Careful selection of the size of the device is crucial, as undersizing may increase the risk of embolisation or failure to seal [3, 10], whereas oversizing can cause rupture of the PTAP [5]. Additionally, a sufficiently narrow neck size is essential given the device diameters currently available [3]. However, the neck of the PTAP must not be too narrow because of the minimum diameters of the delivery catheters. Besides, sufficient rim of tissue in addition to sufficient neck length are indispensable in order to prevent device migration [3, 10]. Finally, it needs to be ensured that there is an adequate distance between the PTAP and adjacent structures in order to prevent occlusion of coronary arteries or great vessels [3, 10].

Previous studies have demonstrated that minimal residual flow is detected in 75% of cases directly postdeployment [7]. Our study reports a 100% rate of minimal or no residual flow on aortography directly postdeployment, corresponding to complete closure of the PTAP in 64% (*n* = 7) and minimal residual flow into the PTAP in 36% (*n* = 4) of cases. However, complete occlusion of the PTAP was present on follow-up CT in only 36% at both early and latest follow-up, whereas partial occlusion was achieved in 36% at early follow-up [2 (IQR 2–3) months] and 9% at latest follow-up [11 (IQR 9–16) months]. The relatively small sample size of this study limited analysis of a potential association between clinical variables at baseline and short or intermediate outcome. A multicentre registry study is required in order to identify the long-term outcome in a larger patient population and to detect predictors of both success and failure of PTAP closure.

Although it is likely that ongoing thrombosis could further occlude the PTAP subsequent to closure device placement, this case series shows that this is not always the case. Theoretically, the perioperative use of high-dose antithrombotic medication could play a role in the development of incomplete sealing of the neck of the PTAP. In our case series, all patients with residual flow on follow-up CT continued their anticoagulants during the periprocedural period. In the absence of a standardised anticoagulation strategy protocol, the periprocedural prescription of anticoagulation therapy was based on the policy of the treating physician and did not depend on the presence of a PTAP. The postoperative prescription of anticoagulation therapy was similarly determined by the preoperative indication (e.g. atrial fibrillation). Of note is that the follow-up strategy (i.e. the time of follow-up CT) was independent of the medical therapy.

Furthermore, residual flow or subtotal occlusion might be explained by inadequate sealing of the neck of the PTAP either because of an insufficiently large diameter of the closure device or because of a spherical or asymmetrical (i.e. non-cylindrical) shape of the neck. Of the cases that demonstrated significant residual flow on follow-up CT, inadequate sealing of the neck of the PTAP could be confirmed by 3D reconstructions for cases 5 and 7. Although no clear association was found between the diameter of the neck and that of the device in our series, this does imply that a larger device diameter would have been necessary to achieve complete sealing in this subset of patients. This highlights the importance of adequate preoperative imaging with the application of 3D reconstructions, in order to identify PTAPs that are suitable for transcatheter closure and to determine the size of closure device that best fits the neck of the PTAP.

## Conclusion

Based on this preliminary experience, we conclude that even though subtotal or total occlusion of the PTAP was seen at early and latest follow-up in only 45–73% of our cases, transcatheter closure can offer a valuable minimally invasive primary treatment option for patients with a high surgical risk. If transcatheter device closure fails, a more radical high-risk reoperation can be performed as a last resort.

The rate of successful occlusion could possibly be further improved by standardised preprocedural 3D image reconstruction and by subsequent patient-specific 3D printing. Given that our findings are based on a limited number of cases, these results should be substantiated by future studies.

### Supplementary Information

Table S1. Procedural information per patient
