# Supplementary material for: Transcatheter closure of postsurgical aortic pseudoaneurysms guided by three-dimensional image reconstruction: a single-centre experience
Source: Neth Heart J. 2023 May 31;31(10):383–9. doi: 10.1007/s12471-023-01784-1 (PMC10516814; doi:10.1007/s12471-023-01784-1)
Supplement: Supplementary file 1 — Table S1. Procedural information per patient [file 12471_2023_1784_MOESM1_ESM.docx]

**Table S1. Procedural information per patient**

| **Case** | **Duration between first surgery and diagnosis (y)** | **Sac: Width (mm)** | **Sac: Depth (mm)** | **Sac: Height (mm)** | **Neck*** | **Device** | **Device size: Long Axis (mm)** | **Device size: Short Axis (mm)** | **Procedural time (min)** | **Contrast (ml)** | **Procedural complications** | **Residual flow after device deployment on aortography** | **Occlusion on short-term follow-up CT** | **Occlusion on last follow-up CT** |
| --- | --- | --- | --- | --- | --- | --- | --- | --- | --- | --- | --- | --- | --- | --- |
| **1** | 5 | 51 | 43 | 68 | 6 | AVP-3 | 10 | 5 | 53 | 320 | None | Minimal | Partial occlusion | Partial occlusion* |
| **2** | 4 | 30 | 40 | 38 | 7 | AVP-3 | 14 | 5 | 52 | 250 | None | None | Occlusion | Occlusion |
| **3** | 8 | 30 | 10 | 25 | 4 | Occlutech ASD Occluder | 15 |  | 116 |  | None | None | Partial occlusion | NA |
| **4** | 5 | 8 | 12 |  | 6 | AVP-3 | 6 | 3 | 59 | 150 | None | None | Occlusion | Occlusion |
| **5** | 8 | 30 | 16 | 26 | 5 | AVP-3 | 6 | 3 | 66 | 220 | None | None | Significant residual flow | Significant residual flow |
| **6** | 4 | 19 | 8 | 16 | 3 | AVP-3 | 8 | 4 | 94 | 250 | None | Minimal | Partial occlusion | Significant residual flow |
| **7** | 27 | 30 | 12 | 23 | 6 | Amplatzer Septal Occluder | 10 |  | 120 | 230 | None | Minimal | Significant residual flow | Significant residual flow |
| **8** | 2 | 38 | 17 | 30 | 6 | AVP-3 | 10 | 5 | 250 | 200 | None | None | Significant residual flow | Significant residual flow |
| **9** | 24 | 22 | 21 | 21 | 12 | AVP-3 | 10 | 5 | 63 | 75 | None | Minimal | Occlusion | Occlusion |
| **10** | 6 | 26 | 15 | 30 | Not visible | AVP-3 | 6 | 3 | 61 | 100 | None | None | Partial occlusion | Occlusion |
| **11** | 9 | 13 | 13 | 16 | 5 | AVP-3  AVP-3 | 8  6 | 4  3 | 141 | 700 | None | None | Occlusion | NA |

Abbreviations: ASD, Atrial Septal Defect; AVP, Amplatzer Valvular Plug; CT, computed tomography; mm, millimeter; NA, not available. *, largest diameter.
